# Redetermination of K_2_Mg_3_(OH)_2_(SO_4_)_3_(H_2_O)_2_ from single-crystal X-ray data revealing the correct hydrogen-atom positions

**DOI:** 10.1107/S2056989020016217

**Published:** 2021-01-01

**Authors:** Matthias Weil

**Affiliations:** a Institute for Chemical Technologies and Analytics, Division of Structural Chemistry, TU Wien, Getreidemarkt 9/164-SC, A-1060 Vienna, Austria

**Keywords:** crystal structure, redetermination, potassium magnesium sulfate, hydrogen bonding, structure comparison

## Abstract

In comparison with the previous structure determination of K_2_Mg_3_(OH)_2_(SO_4_)_3_(H_2_O)_2_, dipotassium trimagnesium di­hydroxide tris­(sulfate) dihydrate, from laboratory powder X-ray diffraction data [Kubel & Cabaret-Lampin (2013 ▸). *Z. Anorg. Allg. Chem.* **639**, 1782–1786], the present redetermination against CCD single-crystal data has allowed for the modelling of all non-H atoms with anisotropic displacement parameters. As well as higher accuracy and precision in terms of bond lengths and angles, the clear localization of the H-atom positions leads also to a reasonable hydrogen-bonding scheme for this hy­droxy hydrate. The structure consists of (100) sheets composed of corner- and edge-sharing [MgO_6_] octa­hedra and sulfate tetra­hedra. Adjacent sheets are linked by the potassium cations and a hydrogen bond of medium strength involving the water mol­ecule. The title compound is isotypic with its Co^II^ and Mn^II^ analogues: the three K_2_ *M*_3_(OH)_2_(SO_4_)_3_(H_2_O)_2_ (*M* = Mg, Co, Mn) structures are qu­anti­tatively compared.

## Abstract 2

A single-crystal X-ray diffraction study of K_2_Mg_3_(OH)_2_(SO_4_)_3_(H_2_O)_2_ revealed the locations of all H atoms and hence a revised hydrogen-bonding scheme in comparison with a previous powder X-ray diffraction study.

## Chemical context   

In our recent projects focused on hydro­thermal phase-formation studies in the systems *M*/*X* ^VI^/Te^IV^/O/H (*X* = S, Se), it was tested whether tetra­hedral sulfate or selenate anions can be incorporated into oxidotellurates(IV) of different divalent metals *M*. So far, this concept proved to be successful for *M* = Hg (Weil & Shirkhanlou, 2015 ▸), *M* = Ca, Cd, Sr (Weil & Shirkhanlou, 2017*a* ▸), *M* = Pb (Weil & Shirkhanlou, 2017*b* ▸) as well as for *M* = Zn, Mg (Weil & Shirkhanlou, 2017*c* ▸). However, in nearly all cases multi-phase formation was observed under the given hydro­thermal conditions, and the target compounds, *i.e.* metal oxidochalcogenates(IV,VI) with both oxido­sulfate­(VI) or oxidoselenate(VI) *and* oxidotellurate(IV) building units, appeared only as minority phases next to other different phases. The same holds for the Mg/S/Te/O/H system when working at pH ∼10 by using potassium hydroxide as a base. From one of the reaction batches, high-quality single crystals of the title compound, K_2_Mg_3_(OH)_2_(SO_4_)_3_(H_2_O)_2_, could be isolated as one of the products. A crystal-structure refinement of this phase has already been performed by Rietveld refinement against laboratory powder X-ray diffraction data (Kubel & Cabaret-Lampin, 2013 ▸). In the corresponding structure model, H-atom positions were estimated and optimized by energy minimization, but the resulting hydrogen-bonding pattern was not discussed in detail. A close check of this model revealed chemically implausible O—H bond lengths and O—H⋯O angles (Table 1 ▸). For example, H1 is more tightly bonded to O7 than to the actual hydroxide O atom (O8); the second hydroxyl group (O9) shows a too large O—H distance accompanied with large *D*⋯*A* distances or a too small O9—H2⋯O8 angle; the water mol­ecule (O10) shows likewise either unreasonable H⋯*A* distances or *D*—H⋯*A* angles. Hence a redetermination of the crystal structure of K_2_Mg_3_(OH)_2_(SO_4_)_3_(H_2_O)_2_ to establish a more reasonable hydrogen-bonding pattern by using single crystal X-ray diffraction CCD data seemed appropriate and is reported here.

## Structural commentary   

**Figure 1:**
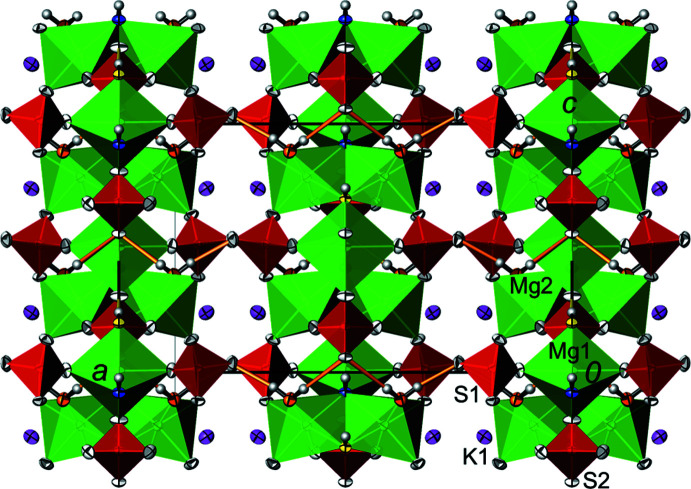
The crystal structure of K_2_Mg_3_(OH)_2_(SO_4_)_3_(H_2_O)_2_ in a projection along [0
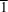
0]. Displacement ellipsoids are drawn at the 98% probability level for non-H atoms, and H atoms are given as spheres of arbitrary radius. Colour code: [MgO_6_] octa­hedra are green, SO_4_ tetra­hedra are red; sulfate O atoms are white, O atoms of OH are yellow (O8) and blue (O9), and O atoms of water mol­ecules are orange (O10). Hydrogen bonds involving the hy­droxy group O8 are indicated by yellow lines and those involving the water mol­ecule by orange lines.

Of the 19 atoms in the asymmetric unit (1 K, 2 Mg, 2 S, 10 O, 4 H), eight (Mg1, S2, O5, O7, O8, O9, H1 and H2) are located on a crystallographic mirror plane at *x* = 0 (Wyckoff position 4 *a*); all other atoms in the asymmetric unit are on general sites (8 *b*). Both Mg^II^ atoms are octa­hedrally coordinated by oxygen atoms. Mg1 is bonded to four O atoms belonging to sulfate groups (O5, O1 and its symmetry-related counterpart, O7) and to O atoms of two OH groups (O8, O9), whereas Mg2 is bonded to three sulfate O atoms (O4, O6, O2), two OH groups (O8, O9) and an O atom belonging to a water mol­ecule (O10). Two [Mg2(H_2_O)(OH)_2_O_3_] octa­hedra build up a {Mg2(H_2_O)_1/1_O_3/1_(OH)_2/2_}_2_ dimer by edge-sharing the two OH groups. These dimers are linked to the [Mg1(OH)_2_O_4_] octa­hedra by corner-sharing the two OH groups, which leads to the formation of zigzag chains running parallel to [001]. Sulfate tetra­hedra join neighbouring chains into sheets extending parallel to (100). Adjacent sheets are linked into a three-dimensional network by potassium cations (irregular nine-coordination), together with a hydrogen bond involving the water mol­ecule (O10) and a sulfate O atom (O3) (Fig. 1 ▸).

The bond lengths for the two octa­hedral [MgO_6_] groups, the tetra­hedral sulfate groups and the nine-coordinate potassium cations, with mean values of 2.089, 1.474 and 2.964 Å, respectively, are in very good agreement with the expected values of 2.089 (59), 1.473 (7) and 2.955 (214) Å, provided recently by Gagné & Hawthorne (2016 ▸, 2018 ▸). In terms of a comparison between the current single-crystal study and the previous powder study by Kubel & Cabaret-Lampin (2013 ▸), individual bond lengths as obtained from the single crystal study are, as expected, more precise and accurate, with the largest deviation of Δ = 0.092 Å for the Mg1—O5^iv^ bond (Table 2 ▸).

The hydrogen-bonding pattern derived from the single crystal study is chemically plausible (Table 3 ▸, Fig. 1 ▸). The water mol­ecule (O10) participates in two nearly linear O—H⋯O hydrogen bonds to two sulfate O atoms. One is of weak nature to O5 as the acceptor atom [*D*⋯*A* = 3.009 (2) Å] within a sheet, the other of medium strength to O3 [*D*⋯*A* = 2.722 (2) Å] between adjacent layers. The hy­droxy group involving O8 exhibits a weak hydrogen bond to a neighbouring sulfate O atom [O7; *D*⋯*A* = 3.068 (2) Å]. The other hy­droxy group involving O9 appears not to be involved in hydrogen bonding: the next nearest O atoms that could act as acceptor atoms are two symmetry-related O6 atoms (−*x*, −*y* + 1, *z* + 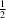; *x*, −*y* + 1, *z* + 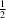), both at a distance of 3.405 (2) Å from O9. Such a long *D*⋯*A* distance is usually not considered as relevant for hydrogen bonding but was discussed for the K_2_Co_3_(OH)_2_(SO_4_)_3_(H_2_O)_2_ structure as part of a bifurcated O—H⋯(O,O) hydrogen bond of very weak nature, here with *D*⋯*A* = 3.370 (9) Å (Effenberger & Langhof, 1984 ▸).

K_2_Mg_3_(OH)_2_(SO_4_)_3_(H_2_O)_2_ is isotypic with its Co (Effenberger & Langhof, 1984 ▸) and Mn (Yu *et al.*, 2007 ▸) analogues. The three isotypic K_2_ *M*_3_(OH)_2_(SO_4_)_3_(H_2_O)_2_ (*M* = Mg, Co, Mn) structures were qu­anti­tatively compared using the *compstru* program (de la Flor *et al.*, 2016 ▸), available at the Bilbao Crystallographic Server (Aroyo *et al.*, 2006 ▸). For this purpose, the hydrogen atoms were not taken into account. In relation to the title Mg structure, the Co and Mn structures show the following values for evaluation of the structural similarity. Co: the degree of lattice distortion is 0.0034, the maximum displacement between atomic positions of paired atoms is 0.0553 Å for the pair O9, the arithmetic mean of the distance between paired atoms is 0.0295 Å, and the measure of similarity is 0.010. Corresponding values for the Mn structure are: 0.0126, 0.1343 Å for pair O2, 0.0768 Å and 0.013, respectively. The two value sets indicate a higher similarity between the Mg and Co structures compared to the Mn structure. This is most probably related to the ionic radii (Shannon, 1976 ▸) of the six-coordinate metal cations that differ only marginally for Mg (0.72 Å) and Co (0.745 Å, assuming a high-spin 3*d* ^7^ configuration), whereas Mn (0.83 Å for a high-spin 3*d* ^5^ state) is considerately greater.

## Synthesis and crystallization   

A mixture of 380 mg of MgSO_4_·7H_2_O, 100 mg of TeO_2_ and 70 mg of KOH was placed in a 5 ml Teflon container that was subsequently filled with 2 ml of water and sealed with a Teflon lid. The closed container was placed in a steel autoclave and was heated at 413 K for one week at autogenous pressure and then cooled down to room temperature within 5 h. The recovered solids consisted of Mg_2_Te_3_O_8_ (Lin *et al.*, 2013 ▸) as the main product (checked by powder X-ray diffraction of the bulk), besides minor amounts of caminite, Mg_2_(SO_4_)(OH)_2_ (Keefer *et al.*, 1981 ▸), the sulfate tellurite Mg_3_(SO_4_)(TeO_3_)(OH)_2_(H_2_O)_2_ (Weil & Shirkhanlou, 2017*c* ▸) and the title compound (the latter phases determined by single-crystal X-ray diffraction).

## Refinement   

Crystal data, data collection and structure refinement details are summarized in Table 4 ▸. Atomic coordinates and labelling for the non-H atoms were adapted from the previous refinement from powder X-ray diffraction data (Kubel & Cabaret-Lampin, 2013 ▸). H atoms were clearly discernible in difference-Fourier maps and were refined freely. The Flack parameter (Table 4 ▸) indicates that the absolute structure has been determined correctly.

## Supplementary Material

Crystal structure: contains datablock(s) I, global. DOI: 10.1107/S2056989020016217/hb7955sup1.cif

Structure factors: contains datablock(s) I. DOI: 10.1107/S2056989020016217/hb7955Isup2.hkl

CCDC reference: 2050138

Additional supporting information:  crystallographic information; 3D view; checkCIF report

## Figures and Tables

**Table 1:** Hydrogen-bond geometry (Å, °) from the previous model based on powder X-ray diffraction data and geometry-optimized H-atom positions (Kubel & Cabaret-Lampin, 2013 ▸)

*D*—H⋯*A*	*D*—H	H⋯*A*	*D*⋯*A*	*D*—H⋯*A*
O7—H1⋯O8^i^	0.82	2.54	3.258 (14)	147
O9—H2⋯O8	1.13	2.18	2.901 (14)	119
O9—H2⋯O6^ii^	1.13	2.52	3.506 (13)	145
O9—H2⋯O6^iii^	1.13	2.52	3.506 (13)	145
O10—H3⋯O5^iv^	0.85	2.41	3.066 (9)	134
O10—H3⋯O6^iv^	0.85	2.55	3.038 (13)	118
O10—H3⋯O1	0.85	2.52	3.229 (9)	142
O10—H4⋯O2^iii^	0.97	2.39	2.812 (8)	106
O10—H4⋯O3^v^	0.97	1.77	2.698 (9)	158

Symmetry codes; (i) *x*, −*y*, *z* + 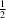; (ii) −*x*, −*y* + 1, *z* + 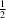; (iii) *x*, −*y* + 1, *z* + 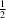; (iv) *x*, *y*, *z* + 1; (v) −*x* + 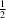, −*y* + 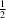, *z* + 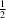.

**Table 2:** Comparison of bond lengths (Å) from the current single-crystal X-ray study and the previous powder X-ray diffraction study (Kubel & Cabaret-Lampin, 2013 ▸)

Bond	single-crystal study	powder study
K1—O4^i^	2.8323 (15)	2.902 (7)
K1—O3^i^	2.8594 (15)	2.877 (8)
K1—O10^ii^	2.8704 (15)	2.874 (9)
K1—O2^iii^	2.9436 (14)	2.948 (8)
K1—O6	2.9743 (15)	2.945 (8)
K1—O3^ii^	2.9915 (15)	3.016 (9)
K1—O2	3.0532 (15)	3.116 (9)
K1—O1^ii^	3.0740 (15)	3.118 (10)
K1—O1	3.0780 (15)	3.128 (11)
K1—O3	3.3068 (15)	3.311 (10)
Mg1—O9	2.067 (2)	2.139 (11)
Mg1—O8	2.071 (2)	2.104 (10)
Mg1—O5^iv^	2.081 (2)	1.989 (10)
Mg1—O1^v^	2.0976 (13)	2.049 (5)
Mg1—O1	2.0977 (13)	2.049 (5)
Mg1—O7	2.160 (2)	2.139 (11)
Mg2—O4	2.0220 (15)	2.012 (9)
Mg2—O6^vi^	2.0796 (14)	2.099 (8)
Mg2—O2^vi^	2.0924 (15)	2.117 (8)
Mg2—O8	2.0952 (14)	2.036 (7)
Mg2—O9^vii^	2.1026 (14)	2.045 (9)
Mg2—O10	2.1064 (15)	2.163 (8)
S1—O4	1.4657 (14)	1.487 (10)
S1—O3	1.4659 (14)	1.463 (6)
S1—O2	1.4818 (14)	1.500 (10)
S1—O1	1.4818 (13)	1.476 (6)
S2—O7	1.469 (2)	1.483 (13)
S2—O6	1.4751 (14)	1.468 (8)
S2—O6^v^	1.4751 (14)	1.468 (8)
S2—O5	1.4779 (19)	1.530 (12)

Symmetry codes: (i) −*x* + 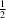, −*y* + 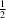, *z* − 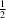; (ii) *x*, −*y*, *z* − 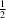; (iii) −*x* + 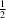, *y* − 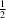, *z*; (iv) −*x*, −*y*, *z* + 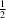; (v) −*x*, *y*, *z*; (vi) *x*, −*y* + 1, *z* + 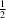; (vii) −*x*, −*y* + 1, *z* + 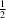.

**Table 3:** Hydrogen-bond geometry (Å, °)

*D*—H⋯*A*	*D*—H	H⋯*A*	*D*⋯*A*	*D*—H⋯*A*
O8—H1⋯O7^i^	0.86 (4)	2.22 (4)	3.068 (2)	168 (3)
O9—H2	0.73 (5)	?	?	?
O10—H3⋯O5^ii^	0.79 (4)	2.22 (4)	3.009 (2)	171 (3)
O10—H3⋯O6^ii^	0.79 (4)	2.59 (3)	3.023 (2)	116 (3)
O10—H4⋯O3^iii^	0.83 (4)	1.90 (4)	2.722 (2)	171 (4)

Symmetry codes: (i) 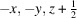; (ii) 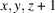; (iii) 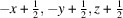.

**Table 4:** Experimental details

Crystal data	
Chemical formula	K_2_Mg_3_(OH)_2_(SO_4_)_3_(H_2_O)_2_
*M*_r_	509.36
Crystal system, space group	Orthorhombic, *C* *m* *c*2_1_
Temperature (K)	100
*a*, *b*, *c* (Å)	17.8228 (19), 7.4879 (8), 9.7686 (10)
*V* (Å^3^)	1303.7 (2)
*Z*	4
Radiation type	Mo *K*α
μ (mm^−1^)	1.45
Crystal size (mm)	0.10 × 0.08 × 0.01

Data collection	
Diffractometer	Bruker APEXII CCD
Absorption correction	Multi-scan (*SADABS*; Krause *et al.*, 2015 ▸)
*T*_min_, *T*_max_	0.668, 0.747
No. of measured, independent and observed [*I* > 2σ(*I*)] reflections	31799, 2535, 2430
*R*_int_	0.042
(sin θ/λ)_max_ (Å^−1^)	0.768

Refinement	
*R*[*F* ^2^ > 2σ(*F* ^2^)], *wR*(*F* ^2^), *S*	0.017, 0.039, 1.07
No. of reflections	2535
No. of parameters	132
No. of restraints	1
H-atom treatment	All H-atom parameters refined
Δρ_max_, Δρ_min_ (e Å^−3^)	0.30, −0.38
Absolute structure	Flack *x* determined using 1125 quotients [(*I* ^+^)−(*I* ^−^)]/[(*I* ^+^)+(*I* ^−^)] (Parsons *et al.*, 2013 ▸)
Absolute structure parameter	−0.010 (13)

Coordinates taken from previous refinement. Computer programs: *APEX3* and *SAINT* (Bruker, 2015 ▸), *SHELXL2014/7* (Sheldrick, 2015 ▸), *ATOMS for Windows* (Dowty, 2006 ▸) and *publCIF* (Westrip, 2010 ▸).

## supplementary crystallographic information

### Crystal data

**Table:**

K_2_Mg_3_(OH)_2_(SO_4_)_3_(H_2_O)_2_	*D*_x_ = 2.595 Mg m^−^^3^
*M**_r_* = 509.36	Mo *K*α radiation, λ = 0.71073 Å
Orthorhombic, *C**m**c*2_1_	Cell parameters from 9959 reflections
*a* = 17.8228 (19) Å	θ = 2.8–33.0°
*b* = 7.4879 (8) Å	µ = 1.45 mm^−^^1^
*c* = 9.7686 (10) Å	*T* = 100 K
*V* = 1303.7 (2) Å^3^	Plate, colourless
*Z* = 4	0.10 × 0.08 × 0.01 mm
*F*(000) = 1024	

### Data collection

**Table:**

Bruker APEXII CCD diffractometer	2430 reflections with *I* > 2σ(*I*)
ω– and φ–scans	*R*_int_ = 0.042
Absorption correction: multi-scan (*SADABS*; Krause *et al.*, 2015)	θ_max_ = 33.1°, θ_min_ = 2.3°
*T*_min_ = 0.668, *T*_max_ = 0.747	*h* = −27→27
31799 measured reflections	*k* = −11→11
2535 independent reflections	*l* = −14→14

### Refinement

**Table:**

Refinement on *F*^2^	Hydrogen site location: difference Fourier map
Least-squares matrix: full	All H-atom parameters refined
*R*[*F*^2^ > 2σ(*F*^2^)] = 0.017	*w* = 1/[σ^2^(*F*_o_^2^) + (0.0167*P*)^2^ + 0.9127*P*] where *P* = (*F*_o_^2^ + 2*F*_c_^2^)/3
*wR*(*F*^2^) = 0.039	(Δ/σ)_max_ < 0.001
*S* = 1.07	Δρ_max_ = 0.30 e Å^−^^3^
2535 reflections	Δρ_min_ = −0.38 e Å^−^^3^
132 parameters	Absolute structure: Flack *x* determined using 1125 quotients [(*I*^+^)-(*I*^-^)]/[(*I*^+^)+(*I*^-^)] (Parsons *et al.*, 2013)
1 restraint	Absolute structure parameter: −0.010 (13)

### Special details

**Table:**

Geometry. All esds (except the esd in the dihedral angle between two l.s. planes) are estimated using the full covariance matrix. The cell esds are taken into account individually in the estimation of esds in distances, angles and torsion angles; correlations between esds in cell parameters are only used when they are defined by crystal symmetry. An approximate (isotropic) treatment of cell esds is used for estimating esds involving l.s. planes.

### Fractional atomic coordinates and isotropic or equivalent isotropic displacement parameters (Å^2^)

**Table:**

	*x*	*y*	*z*	*U*_iso_*/*U*_eq_	
K1	0.19321 (2)	0.04648 (5)	−0.26016 (4)	0.00947 (7)	
Mg1	0.0000	0.19893 (11)	0.00487 (10)	0.00514 (15)	
Mg2	0.08605 (4)	0.46648 (8)	0.28489 (7)	0.00532 (11)	
S1	0.17492 (2)	0.30375 (5)	0.01513 (5)	0.00452 (7)	
S2	0.0000	0.18319 (8)	−0.31682 (6)	0.00448 (10)	
O1	0.11670 (7)	0.16279 (16)	0.00789 (16)	0.0074 (2)	
O2	0.17476 (8)	0.40504 (19)	−0.11519 (14)	0.0075 (2)	
O3	0.24837 (8)	0.21941 (17)	0.03462 (15)	0.0091 (2)	
O4	0.15996 (8)	0.42187 (18)	0.13162 (14)	0.0079 (2)	
O5	0.0000	0.0697 (2)	−0.44063 (19)	0.0067 (3)	
O6	0.06775 (8)	0.29624 (17)	−0.32058 (15)	0.0086 (2)	
O7	0.0000	0.0711 (2)	−0.1934 (2)	0.0087 (4)	
O8	0.0000	0.3133 (2)	0.1979 (2)	0.0055 (3)	
O9	0.0000	0.4505 (2)	−0.0823 (2)	0.0062 (3)	
O10	0.12236 (8)	0.23658 (19)	0.39003 (15)	0.0084 (2)	
H1	0.0000	0.213 (6)	0.241 (5)	0.023 (11)*	
H2	0.0000	0.509 (7)	−0.023 (5)	0.031 (13)*	
H3	0.092 (2)	0.182 (5)	0.433 (3)	0.030 (9)*	
H4	0.159 (2)	0.246 (6)	0.441 (4)	0.046 (12)*	

### Atomic displacement parameters (Å^2^)

**Table:**

	*U*^11^	*U*^22^	*U*^33^	*U*^12^	*U*^13^	*U*^23^
K1	0.01028 (16)	0.00894 (14)	0.00920 (15)	−0.00051 (13)	0.00151 (15)	−0.00034 (14)
Mg1	0.0061 (4)	0.0045 (3)	0.0048 (4)	0.000	0.000	0.0002 (3)
Mg2	0.0054 (3)	0.0052 (3)	0.0053 (2)	0.00037 (19)	0.0001 (2)	−0.0003 (2)
S1	0.00477 (16)	0.00458 (15)	0.00422 (16)	0.00040 (13)	−0.00005 (15)	0.00002 (15)
S2	0.0054 (2)	0.0040 (2)	0.0041 (2)	0.000	0.000	−0.00057 (19)
O1	0.0063 (5)	0.0060 (5)	0.0101 (6)	−0.0022 (4)	0.0003 (5)	−0.0007 (5)
O2	0.0087 (6)	0.0093 (6)	0.0044 (6)	−0.0004 (5)	−0.0007 (4)	0.0021 (4)
O3	0.0057 (5)	0.0100 (6)	0.0114 (6)	0.0025 (4)	0.0006 (5)	0.0013 (5)
O4	0.0092 (6)	0.0084 (6)	0.0061 (6)	−0.0016 (5)	0.0022 (5)	−0.0028 (4)
O5	0.0087 (8)	0.0062 (8)	0.0053 (8)	0.000	0.000	−0.0021 (6)
O6	0.0082 (6)	0.0082 (5)	0.0096 (6)	−0.0037 (4)	0.0018 (5)	−0.0028 (5)
O7	0.0144 (9)	0.0055 (8)	0.0061 (8)	0.000	0.000	0.0009 (6)
O8	0.0059 (8)	0.0048 (7)	0.0056 (8)	0.000	0.000	0.0006 (6)
O9	0.0081 (8)	0.0049 (7)	0.0056 (8)	0.000	0.000	−0.0005 (6)
O10	0.0077 (6)	0.0084 (6)	0.0093 (6)	−0.0006 (5)	−0.0008 (5)	0.0020 (5)

### Geometric parameters (Å, º)

**Table:**

K1—O4^i^	2.8323 (15)	Mg2—O4	2.0220 (15)
K1—O3^i^	2.8594 (15)	Mg2—O6^vi^	2.0796 (14)
K1—O10^ii^	2.8704 (15)	Mg2—O2^vi^	2.0924 (15)
K1—O2^iii^	2.9436 (14)	Mg2—O8	2.0952 (14)
K1—O6	2.9743 (15)	Mg2—O9^vii^	2.1026 (14)
K1—O3^ii^	2.9915 (15)	Mg2—O10	2.1064 (15)
K1—O2	3.0532 (15)	S1—O4	1.4657 (14)
K1—O1^ii^	3.0740 (15)	S1—O3	1.4659 (14)
K1—O1	3.0780 (15)	S1—O2	1.4818 (14)
K1—O3	3.3068 (15)	S1—O1	1.4818 (13)
Mg1—O9	2.067 (2)	S2—O7	1.469 (2)
Mg1—O8	2.071 (2)	S2—O6	1.4751 (14)
Mg1—O5^iv^	2.081 (2)	S2—O6^v^	1.4751 (14)
Mg1—O1^v^	2.0976 (13)	S2—O5	1.4779 (19)
Mg1—O1	2.0977 (13)	O10—H3	0.79 (4)
Mg1—O7	2.160 (2)	O10—H4	0.83 (4)
			
O4^i^—K1—O3^i^	49.50 (4)	O4—Mg2—O9^vii^	168.75 (7)
O4^i^—K1—O10^ii^	131.23 (4)	O6^vi^—Mg2—O9^vii^	86.48 (7)
O3^i^—K1—O10^ii^	166.22 (4)	O2^vi^—Mg2—O9^vii^	97.32 (6)
O4^i^—K1—O2^iii^	58.07 (4)	O8—Mg2—O9^vii^	83.00 (6)
O3^i^—K1—O2^iii^	105.47 (4)	O4—Mg2—O10	91.48 (6)
O10^ii^—K1—O2^iii^	80.80 (4)	O6^vi^—Mg2—O10	171.10 (7)
O4^i^—K1—O6	124.61 (4)	O2^vi^—Mg2—O10	85.19 (6)
O3^i^—K1—O6	75.47 (4)	O8—Mg2—O10	88.59 (7)
O10^ii^—K1—O6	103.59 (4)	O9^vii^—Mg2—O10	99.50 (7)
O2^iii^—K1—O6	157.33 (4)	O4—S1—O3	108.76 (8)
O4^i^—K1—O3^ii^	60.13 (4)	O4—S1—O2	110.97 (8)
O3^i^—K1—O3^ii^	79.54 (4)	O3—S1—O2	109.50 (8)
O10^ii^—K1—O3^ii^	89.75 (4)	O4—S1—O1	109.84 (8)
O2^iii^—K1—O3^ii^	79.64 (4)	O3—S1—O1	108.94 (8)
O6—K1—O3^ii^	122.18 (4)	O2—S1—O1	108.80 (8)
O4^i^—K1—O2	101.48 (4)	O7—S2—O6	110.39 (7)
O3^i^—K1—O2	79.94 (4)	O7—S2—O6^v^	110.39 (7)
O10^ii^—K1—O2	111.40 (4)	O6—S2—O6^v^	109.88 (11)
O2^iii^—K1—O2	100.33 (4)	O7—S2—O5	110.07 (11)
O6—K1—O2	57.19 (4)	O6—S2—O5	108.03 (7)
O3^ii^—K1—O2	158.66 (4)	O6^v^—S2—O5	108.03 (7)
O4^i^—K1—O1^ii^	100.22 (4)	S1—O1—Mg1	127.09 (8)
O3^i^—K1—O1^ii^	87.55 (4)	S1—O1—K1^viii^	90.99 (6)
O10^ii^—K1—O1^ii^	78.76 (4)	Mg1—O1—K1^viii^	121.06 (6)
O2^iii^—K1—O1^ii^	121.72 (4)	S1—O1—K1	86.09 (6)
O6—K1—O1^ii^	80.84 (4)	Mg1—O1—K1	117.63 (7)
O3^ii^—K1—O1^ii^	46.57 (4)	K1^viii^—O1—K1	106.63 (4)
O2—K1—O1^ii^	137.93 (4)	S1—O2—Mg2^ix^	129.61 (8)
O4^i^—K1—O1	134.68 (4)	S1—O2—K1^x^	126.58 (7)
O3^i^—K1—O1	125.75 (4)	Mg2^ix^—O2—K1^x^	102.37 (5)
O10^ii^—K1—O1	65.12 (4)	S1—O2—K1	87.03 (6)
O2^iii^—K1—O1	92.68 (4)	Mg2^ix^—O2—K1	105.63 (5)
O6—K1—O1	69.99 (4)	K1^x^—O2—K1	90.41 (4)
O3^ii^—K1—O1	154.68 (4)	S1—O3—K1^xi^	98.75 (7)
O2—K1—O1	46.28 (3)	S1—O3—K1^viii^	94.61 (7)
O1^ii^—K1—O1	125.08 (4)	K1^xi^—O3—K1^viii^	93.32 (4)
O4^i^—K1—O3	91.01 (4)	S1—O3—K1	77.88 (6)
O3^i^—K1—O3	105.201 (16)	K1^xi^—O3—K1	163.50 (5)
O10^ii^—K1—O3	88.56 (4)	K1^viii^—O3—K1	103.02 (4)
O2^iii^—K1—O3	58.95 (4)	S1—O4—Mg2	142.48 (9)
O6—K1—O3	98.63 (4)	S1—O4—K1^xi^	99.91 (7)
O3^ii^—K1—O3	138.28 (5)	Mg2—O4—K1^xi^	108.16 (6)
O2—K1—O3	44.26 (3)	S2—O5—Mg1^xii^	139.91 (12)
O1^ii^—K1—O3	166.75 (4)	S2—O6—Mg2^ix^	127.33 (9)
O1—K1—O3	43.96 (4)	S2—O6—K1	104.45 (7)
O9—Mg1—O8	89.91 (8)	Mg2^ix^—O6—K1	108.72 (6)
O9—Mg1—O5^iv^	170.48 (9)	S2—O7—Mg1	118.84 (11)
O8—Mg1—O5^iv^	99.61 (8)	Mg1—O8—Mg2	126.57 (6)
O9—Mg1—O1^v^	97.08 (4)	Mg1—O8—Mg2^v^	126.57 (6)
O8—Mg1—O1^v^	92.32 (5)	Mg2—O8—Mg2^v^	94.11 (8)
O5^iv^—Mg1—O1^v^	82.63 (4)	Mg1—O8—H1	95 (3)
O9—Mg1—O1	97.08 (4)	Mg2—O8—H1	106.4 (19)
O8—Mg1—O1	92.32 (5)	Mg2^v^—O8—H1	106.4 (19)
O5^iv^—Mg1—O1	82.63 (4)	Mg1—O9—Mg2^xiii^	121.58 (6)
O1^v^—Mg1—O1	165.09 (8)	Mg1—O9—Mg2^ix^	121.58 (6)
O9—Mg1—O7	91.97 (8)	Mg2^xiii^—O9—Mg2^ix^	93.68 (8)
O8—Mg1—O7	178.12 (9)	Mg1—O9—H2	103 (4)
O5^iv^—Mg1—O7	78.51 (8)	Mg2^xiii^—O9—H2	108 (3)
O1^v^—Mg1—O7	87.44 (5)	Mg2^ix^—O9—H2	108 (3)
O1—Mg1—O7	87.44 (5)	Mg2—O10—K1^viii^	119.30 (6)
O4—Mg2—O6^vi^	82.90 (6)	Mg2—O10—H3	118 (3)
O4—Mg2—O2^vi^	85.94 (6)	K1^viii^—O10—H3	101 (2)
O6^vi^—Mg2—O2^vi^	87.52 (6)	Mg2—O10—H4	118 (3)
O4—Mg2—O8	94.94 (6)	K1^viii^—O10—H4	92 (3)
O6^vi^—Mg2—O8	98.73 (7)	H3—O10—H4	105 (4)
O2^vi^—Mg2—O8	173.74 (7)		

Symmetry codes: (i) −*x*+1/2, −*y*+1/2, *z*−1/2; (ii) *x*, −*y*, *z*−1/2; (iii) −*x*+1/2, *y*−1/2, *z*; (iv) −*x*, −*y*, *z*+1/2; (v) −*x*, *y*, *z*; (vi) *x*, −*y*+1, *z*+1/2; (vii) −*x*, −*y*+1, *z*+1/2; (viii) *x*, −*y*, *z*+1/2; (ix) *x*, −*y*+1, *z*−1/2; (x) −*x*+1/2, *y*+1/2, *z*; (xi) −*x*+1/2, −*y*+1/2, *z*+1/2; (xii) −*x*, −*y*, *z*−1/2; (xiii) −*x*, −*y*+1, *z*−1/2.

### Hydrogen-bond geometry (Å, º)

**Table:**

*D*—H···*A*	*D*—H	H···*A*	*D*···*A*	*D*—H···*A*
O8—H1···O7^iv^	0.86 (4)	2.22 (4)	3.068 (2)	168 (3)
O9—H2	0.73 (5)	?	?	?
O10—H3···O5^xiv^	0.79 (4)	2.22 (4)	3.009 (2)	171 (3)
O10—H3···O6^xiv^	0.79 (4)	2.59 (3)	3.023 (2)	116 (3)
O10—H4···O3^xi^	0.83 (4)	1.90 (4)	2.722 (2)	171 (4)

Symmetry codes: (iv) −*x*, −*y*, *z*+1/2; (xi) −*x*+1/2, −*y*+1/2, *z*+1/2; (xiv) *x*, *y*, *z*+1.
